# Risk of heparinoid use in cosmetics and moisturizers in individuals vaccinated against severe acute respiratory syndrome coronavirus 2

**DOI:** 10.1186/s12959-021-00320-8

**Published:** 2021-09-16

**Authors:** Kenji Yamamoto

**Affiliations:** Department of Cardiovascular Surgery, Center of Varicose Veins, Okamura Memorial Hospital, 293-1 Kakita Shimizu-cho, Sunto-gun Shizuoka, Japan

**Keywords:** heparinoid, heparin-induced thrombocytopenia, thromboembolism, vaccine, coronavirus disease, severe acute respiratory syndrome coronavirus 2, COVID-19

## Abstract

Recently, heparin-induced thrombocytopenia (HIT) after vaccination with the vaccines manufactured by AstraZeneca and Pfizer–BioNTech has been published in the *New England Journal of Medicine*. These reports state that heparin was not used around the vaccination period in all cases. HIT after vaccination is more common in women; thus, heparinoid use can be suspected to induce HIT.

Dear Editor,

There has been an increase in the number of severe cases of coronavirus disease 2019 (COVID-19) even with an increase in the number of vaccinated individuals worldwide. Reports on deaths due to thromboembolism or bleeding in the days following vaccination are increasing. Some mRNA vaccines injected into the muscle tissue may persist at the injection site, whereas others may be transported to the bloodstream, some of which may be taken up by vascular endothelial cells. Adenoviral vectors may similarly infect vascular endothelial cells. Host cells invaded by mRNA or viral vectors produce the severe acute respiratory syndrome coronavirus 2 (SARS-CoV-2) spike protein and thus can be attacked by immune cells and newly generated antibodies triggered as a response, leading to necrotic cell death. If vascular endothelial cells are targeted, the risk of thromboembolism and bleeding is increased owing to partial endothelial cell damage and vascular wall disruption, although this is very rare. In addition, the spike protein alone can damage the endothelium, which is manifested by the impaired mitochondrial function and endothelial nitric oxide synthase (eNOS) activity but increased glycolysis [1].

Recently, heparin-induced thrombocytopenia (HIT) after vaccination with the vaccines manufactured by AstraZeneca and Pfizer–BioNTech has been published in the *New England Journal of Medicine* [2, 3]. HIT after vaccination against COVID-19 is called vaccine-induced immune thrombotic thrombocytopenia (VITT). The incidence of VITT is unknown, but it appears to be very rare. Most reports have described a small number of cases among tens of millions of vaccinated individuals [4, 5]. These reports state that heparin, an anticoagulant, was not used around the vaccination period in all cases. The mechanism by which vaccines induce the formation of new antibodies and immune stimulation of pre-existing antibodies is unknown. The binding of vaccine components (viral proteins and free DNA) to PF4 to produce a neoantigen has been suggested as a possible cause [4, 5]. However, considering the mechanism of HIT, it is necessary to consider other possibilities.

HIT is the development of thrombocytopenia due to the administration of various forms of heparin. Initial reports suggest HIT after vaccination is more common in women [4]. However, in a series of 220 definite and probable cases from the United Kingdom, there was no sex preponderance [6]. According to the International Society on Thrombosis and Haemostasis, the men to women ratio is 0.86. If there is actual female predominance, this would be consistent with the epidemiology of the related syndrome of HIT, which affects females slightly more than males (approximately 60–65 % females) [7]. Furthermore, women tend to be prescribed heparinoids more often than men [8]. Thus, heparinoid use can be suspected to induce HIT [9]. Heparinoids are glycosaminoglycans (heparin derivatives) that are widely used in cosmetics and moisturizers. These may be unintentionally prescribed as a moisturizer for dermatitis. In Japan, heparinoids are covered by insurance as a prescription drug, and their widespread use has become a social issue [8]. It is also marketed as an over-the-counter drug. Furthermore, the drug information states that heparinoids should not be used in thrombocytopenia cases. The daily use of heparinoids may lead to HIT via transdermal absorption [10].

Although determining the degree of heparinoid use in individual countries is difficult, it is necessary to check for the possibility of HIT when administering vaccines and avoid heparinoid use as a preventive measure. Further research on the following is needed to confirm this hypothesis: the difference in the HIT rate between women who use heparinoid and those who do not and how long should heparinoid not be used before receiving vaccination.

For those who cannot be vaccinated, such as individuals with a history of anaphylaxis or those who already have HIT antibodies due to continuous heparinoid use, nonvaccination-dependent measures to prevent the exacerbation of COVID-19 have been reported [11].

In conclusion, the use of heparinoids may be a factor to be considered in the mechanism of VITT.

## Data Availability

Not applicable.

## Abbreviations

HIT: Heparin-induced thrombocytopenia
VITT: Vaccine-induced immune thrombotic thrombocytopenia
